# The Gut Microbiotassay: a high-throughput qPCR approach combinable with next generation sequencing to study gut microbial diversity

**DOI:** 10.1186/1471-2164-14-788

**Published:** 2013-11-14

**Authors:** Marie Louise Hermann-Bank, Kerstin Skovgaard, Anders Stockmarr, Niels Larsen, Lars Mølbak

**Affiliations:** 1 Section for Bacteriology, Pathology and Parasitology, National Veterinary Institute, Technical University of Denmark, Bülowsvej 27, 1870 Frederiksberg C, Denmark; 2 Section for immunology and vaccinology, National Veterinary Institute, Technical University of Denmark, Bülowsvej 27, 1870 Frederiksberg C, Denmark; 3 Department of Informatics and Mathematical Modelling, Technical University of Denmark, Richard Petersens Plads, Building 305, room 126, 2800 Lyngby, Denmark; 4 Danish Genome Institute, Skt. Lucas Kirkeplads 8, 8000 Århus, Denmark; 5 Present address: Chr. Hansen, Bøge Allé 10, 2970 Hørsholm, Denmark

**Keywords:** Access Array 48.48, Bacteria, Intestine, Microbiota, qPCR

## Abstract

**Background:**

The intestinal microbiota is a complex and diverse ecosystem that plays a significant role in maintaining the health and well-being of the mammalian host. During the last decade focus has increased on the importance of intestinal bacteria. Several molecular methods can be applied to describe the composition of the microbiota. This study used a new approach, the Gut Microbiotassay: an assembly of 24 primer sets targeting the main phyla and taxonomically related subgroups of the intestinal microbiota, to be used with the high-throughput qPCR chip ‘Access Array 48.48′, AA48.48, (Fluidigm®) followed by next generation sequencing. Primers were designed if necessary and all primer sets were screened against DNA extracted from pure cultures of 15 representative bacterial species. Subsequently the setup was tested on DNA extracted from small and large intestinal content from piglets with and without diarrhoea. The PCR amplicons from the 2304 reaction chambers were harvested from the AA48.48, purified, and sequenced using 454-technology.

**Results:**

The Gut Microbiotassay was able to detect significant differences in the quantity and composition of the microbiota according to gut sections and diarrhoeic status. 454-sequencing confirmed the specificity of the primer sets. Diarrhoea was associated with a reduced number of members from the genus *Streptococcus,* and in particular *S. alactolyticus.*

**Conclusion:**

The Gut Microbiotassay provides fast and affordable high-throughput quantification of the bacterial composition in many samples and enables further descriptive taxonomic information if combined with 454-sequencing.

## Background

Immediately after birth the mammalian gastrointestinal tract is colonized by a complex and diverse microbial ecosystem. The bacterial invasion and the following gut microbial composition has an enormous impact on its host’s health and well-being [1]. To gain a better understanding of this complex ecosystem, culture-independent methods are essential, as a considerable fraction of the intestinal microbiota has not yet been cultured [2]. One of the ongoing controversies is how to study the bacterial composition in complex ecosystems. To date, some of the widely used approaches to characterize the intestinal microbiota are: metagenomics, phylogenetic microarrays, DNA fingerprinting techniques, and qPCR [2-5]. These methods provide different degrees of taxonomic as well as quantitative information on the microbiota [5]. Nonetheless, variation in technical procedures and differences in data treatment and interpretation makes it challenging to compare results between studies. Also, the expenses and time consumption in relation to labour intensity and data analysis vary greatly. Especially metagenomic approaches are receiving increased attention in the study of microbial communities as a result of their shorter sequencing speed, extended read length, and lower costs [5,6]. However, the enormous amount of data generated becomes cumbersome to analyse, and requires lots of dedicated time as well as expertise to manage [6]. The Access Array 48.48, AA48.48, (Fluidigm Corporation, South San Francisco, CA, USA) creates an affordable link between high-throughput qPCR and next generation sequencing (NGS) and provides manageable data with valuable quantitative and taxonomic information.

Since the 1990’s, qPCR has been applied widely due to its quantitative precision, high specificity and sensitivity, broad dynamic range, good reproducibility, and relatively low costs [7]. In general, qPCR is quick to perform with a low to medium throughput, since most qPCR platforms have a capability format of 96 or 384 [7]. The high-throughput qPCR chip AA48.48 combines 48 detector inlets and 48 sample inlets by interconnecting channels into 2304 individual reaction chambers (singleplex) http://www.fluidigm.com/access-array-system.html. In contrast to most qPCR platforms which require reaction volumes between 5 to 100 μl [7], the AA48.48 operates with a reaction volume of approximately 35 nl [8]. By tagging primers at their 5′-end (using the ‘The Access Array 4-primer amplicon tagging strategy’ [8]), and including a 454 Barcode Library (454BL) with the samples, amplicon generation and library preparation is achieved in the same reaction. Afterwards, amplicons can be harvested directly from the AA48.48 sample inlets where the respective samples were initially loaded. The unique barcodes and specific primers enable segregation of the pooled samples in NGS analysis later on. The AA48.48 process time from start to finish is approximately five hours. After each qPCR run Fluidigm Real-Time PCR Analysis software (Fluidigm Corporation) generates a heatmap constructed as a grid of 48 × 48 small squares presenting all the 2304 primer sample combinations. The heatmap depicts the Cq-value for each reaction according to a colour scale, providing a good overview of each sample and the possibility to compare the bacterial profiles across all the samples.

This study describes the design, optimization, and validation of the Gut Microbiotassay: a primer setup consisting of 24 primer sets targeting the main bacterial phyla of the intestinal microbiota at various taxonomic levels, to be used with the AA48.48 in combination with NGS. Furthermore, it demonstrates the applicability of the Gut Microbiotassay on luminal content collected from the small and large intestine of piglets of different diarrhoeic status. Finally, it validates the specificity of the Gut Microbiotassay by sequencing these amplicons. To our knowledge, the AA48.48 has not previously been used to investigate microbial communities.

## Methods

### Development of the Gut Microbiotassay

#### ***Primer design***

Inspired by Rajilic-Stojanovic et al. [9], the primer setup was designed to target the ribosomal RNA genes (16S or 23S) of the major bacterial groups present in the mammalian intestinal microbiota, including the phyla: Firmicutes, Bacteroidetes, Proteobacteria, and Actinobacteria [10]. To gain insight into the bacterial composition, different taxonomic levels were represented: domain, phylum, class, family, genus, and species-specific primers (Table 1).

**Table 1:** Target-specific primer sequences constituting the Gut Microbiotassay in order to target main bacterial phyla and bacterial groups and species of high interest in the mammalian intestine

**Main target**		**rRNA gene**	**Sequence (5′ → 3′)**	***E. coli *** **position**	**Size, bp**	**Reference**	**Reference bacterium**
Domain Bacteria A V2-V3	*Forward*	16S	AGAGTTTGATCCTGGCTCAG	7	336	Liu *et al.*[30]	*1,2,3,4,5,6,7,8,9,10,11,13,14,15*
	*Reverse*	16S	CTGCTGCCTYCCGTA	342		Liu *et al.*[30]	
Domain Bacteria B V4-V5	*Forward*	16S	CAGCAGCCGCGGTAATAC	518	389	Schwieger & Tebbe [31]	*1,2,3,4,5,6,7,8,9,10,11,13,14,15*
	*Reverse*	16S	CCGTCAATTCCTTTGAGTTT	906		Schwieger & Tebbe [31]	
Phylum Firmicutes	*Forward*	16S	CTGATGGAGCAACGCCGCGT	385	429	Haakensen *et al.*[32]	*6,7,9,11,13,14*
	*Reverse*	16S	ACACYTAGYACTCATCGTTT	813		Mühling *et al.*[33]	
Class Bacilli	*Forward*	16S	GCAGTAGGGAATCTTCCGC	353	461	Felske *et al.*[34]	*7,11,14*
	*Reverse*	16S	ACACTTAGCACTCATCGTTT	813		Modified from Mühling *et al.*[33]	
Genus *Enterococcus*	*Forward*	16S	GGGTAACCTRCCCATCAGA	125	325	Modified from Behr *et al.*[35]	*7*
	Reverse*	16S 16S	GTTACTCTCATCCTTGTTC ACCGTCAGGGGACGTTCAG	449 466	342	Modified from Behr *et al.*[35] Modified from Behr *et al.*[35]	
Genus *Lactobacillus*	*Forward*	23S	GCGGTGAAATTCCAAACG	774	216	This study, Linux ARB [13]	*11*
	*Reverse*	23S	GGGACCTTAACTGGTGAT	989		This study, Linux ARB [13]	
Genus *Streptococcus*	*Forward*	16S	CTWACCAGAAAGGGACGGCT	488	337	This study, ClustalW2 [36]	*14*
	*Reverse*	16S	AAGGRYCYAACACCTAGC	824		This study, ClustalW2 [36]	
Family Clostridium cluster I	*Forward*	16S	AAAGGAAGATTAATACCGCATA	159	538	Modified from Rinttila *et al.*[37]	*6*
	*Reverse*	16S	TTCTTCCTAATCTCTACGCA	696		Hung *et al.*[38]	
Species *Clostridium perfringens*	*Forward*	16S	TGAAAGATGGCATCATCATTCAAC	183	258	Skånseng *et al.*[39]	*6*
	*Reverse*	16S	GGTACCGTCATTATCTTCCCCAAA	440		Skånseng *et al.*[39]	
Family Clostridium cluster IV	*Forward*	16S	ACAATAAGTAATCCACCTGG	866	298	Modified from Ramirez-Farias *et al.*[40]	*9*
	*Reverse*	16S	CTTCCTCCGTTTTGTCAA	1163		Matsuki *et al.*[41]	
Family Clostridium cluster XIV	*Forward*	16S	CGGTACCTGACTAAGAAGC	482	413	Rinttila *et al.*[37]	*13*
	*Reverse*	16S	CTTTGAGTTTCATTCTTGCGAA	894		Matsuki *et al.*[42]	
Phylum Bacteroidetes	*Forward*	16S	CCGGAWTYATTGGGTTTAAAGGG	554	414	Mühling *et al.*[33]	*1*
	*Reverse*	16S	GGTAAGGTTCCTCGCGTA	967		Mühling *et al.*[33]	
Genus *Bacteroides*	*Forward*	16S	AAGGTCCCCCACATTGG	302	300	Manz *et al.*[43]	*1*
	*Reverse*	16S	GAGCCGCAAACTTTCACAA	601		Franks *et al.*[44]	
Phylum Actinobacteria	*Forward*	16S	GCGKCCTATCAGCTTGTT	235	333	Modified from Glockner *et al.*[45] first published. They refer to Erhart [46]	*2*
	*Reverse*	16S	CCGCCTACGAGCYCTTTACGC	567		This study, ClustalW2 [36]	
Family Bifidobacteriaceae	*Forward*	16S	CTCCTGGAAACGGGTGG	152	442	Matsuki *et al.*[42]	*2*
	*Reverse*	16S	CTTTCACACCRGACGCG	593		Delroisse *et al.*[47]	
Class β- and γ-proteobacteria	*Forward*	23S	GTATAATGGGTCAGCGAC	569	673	This study, Linux ARB [13]	*8*
	*Reverse*	23S	CAGCATTCGCACTTCTGA	1241		This study, Linux ARB [13]	
Family Enterobacteriacea	*Forward*	16S	CGTCGCAAGMMCAAAGAG	182	333	Modified from Friedrich *et al.*[48]	*8*
	*Reverse*	16S	TTACCGCGGCTGCTGGCAC	514		Modified from Palmer *et al.*[49]	
Species *Escherichia coli*	*Forward*	16S	GTTAATACCTTTGCTCATTGA	461	320	Malinen *et al.*[50]	*8*
	*Reverse*	16S	ACCAGGGTATCTAATCCTGTT	780		Malinen *et al.*[50]	
Class ϵ-proteobacteria	*Forward*	16S	TGGTGTAGGGGTAAAATCCG	680	286	Bui *et al.*[51]	*5*
	*Reverse*	16S	AGGTAAGGTTCTTCGYGTATC	965		This study, Primrose [14]	
Class δ-proteobacteria	*Forward*	16S	GGTGTAGGAGTGAARTCCGT	681	534	This study, Primrose [14]	*3*
	*Reverse*	16S	TACGTGTGTAGCCCTRGRC	1214		This study, Primrose [14]	
Phylum Fusobacteria	*Forward*	16S	GATCCAGCAATTCTGTGTGC	387	292	Sanguin *et al.*[52]	*10*
	*Reverse*	16S	CGAATTTCACCTCTACACTTGT	678		Walter *et al.*[53]	
Phylum Verrucomicrobia	*Forward*	16S	GAATTCTCGGTGTAGCA	673	551	Modified from Ranjan [54]	*15*
	*Reverse*	16S	GGCATTGTAGTACGTGTGCA	1223		This study, Primrose [14]	
Phylum Spirochaetes	*Forward*	16S	GTYTTAAGCATGCAAGTC	45	294	Choi *et al.*[55]	*4*
	*Reverse*	16S	TGCTGCCTCCCGTAGGAG	338		This study, ClustalW2 [36]	
Domain Archaea	*Forward*	16S	CAGCMGCCGCGGTAATWC	518	440	Giovannoni *et al.*[56]	*12*
	*Reverse*	16S	YCCGGCGTTGAMTCCAATT	957		Delong [57]	

Reference bacteria listed are target for the respective primer sets. Numbers represent: ***1:*** *Bacteroides fragilis* (DJF_B083(EU728706)), ***2:*** *Bifidobacterium pseudolongum globosum* (DMS 20092), ***3:*** *Bilophila wadsworthia* (ATCC 49260), ***4:*** *Brachyspira pilosicoli* (isolated from the intestine of slaughter pig at DTU-VET, 28-02-2000), ***5:*** *Campylobacter fetus* (ATTC 10852), ***6:*** *Clostridium perfringens* (NCTC 10240), ***7:*** *Enterococcus faecalis* (ATCC 29212), ***8:*** *Escherichia coli* (9711108–2), ***9:*** *Faecalibacterium prausnitzii* (DSM 17677), ***10:*** *Fusobacterium Necrophorum* (ATCC 25286), ***11:*** *Lactobacillus sakei* (DSM 20017), ***12:*** *Methanocorpusculum labreanum* (DSM 4855), ***13:*** *Roseburia* sp. (DJF_VR77(EU728794)), ***14:*** *Streptococcus suis* (NCTC 10446), and ***15:*** *Verrucomicrobium spinosum* (DSM 4136). Nucleotide explanation: *Y*, C/T ; *R*, A/G ; *W*, A/T ; *K*, G/T ; *M*, A/C.

*To improve coverage of the Genus *Enterococcus*, two reverse primers were mixed in equal concentrations.

Primer specificity was checked *in silico* with the ‘probe match’ facility of the Ribosomal Database Project, release 10 (http://rdp.cme.msu.edu/) [11] and the ‘check’ application of ProbeCheck (http://131.130.66.200/cgi-bin/probecheck/content.pl?id=home) [12] using the programs’ default settings. Judged by the search results from these online databases, primers were renamed according to their main target. Some of the published primers were slightly modified to improve their specificity. New primers were designed using the ‘Probe Design Tool’ of the ARB Software package (http://www.arb-home.de/) [13], or Primrose [14] using default options, and further validated based on BLAST search (NCBI). Primers were designed to have approximately same length of nucleotides, GC-content, a minimum number of degenerate bases, and to produce amplicons between 200–500 bp long, compatible with the read length of the 454 GS FLX Titanium Sequencer. Similar properties were important, given that all the primers had to function under the same conditions when run on an AA48.48.

All primers, except for the species-specific ones, were tagged at their 5′-end to enable incorporation of a 454BL necessary for 454-sequencing: forward tag: 5′-ACACTGACGACATGGTTCTACA-3′, and reverse tag: 5′-TACGGTAGCAGAGACTTGGTCT-3′ in accordance to the ‘Access Array™ System User Guide’ [8]. Primers were purchased from Eurofins MWG Synthesis GmbH (Ebersberg, Germany) and stored at -20°C.

#### ***Empirical testing of primers***

All primers were tested against 15 strains of pure-cultured reference bacteria, representing targets for one or more of the different primer sets (Table 1). Thus the reference bacterial DNA functioned as both positive and negative controls for the individual primer sets.

##### 

**DNA extraction** Chromosomal DNA from cultured reference bacteria (Additional file 1: Table S1) was isolated using the Easy-DNA™ Kit (Invitrogen, Carlsbad, CA, USA), for further details on the DNA extraction protocol see Additional file 2. DNA was precipitated with ethanol, and resuspended in 60 μl TE buffer. The reference bacteria were cultured as recommended by DSMZ (http://www.dsmz.de/).

An interplate calibrator (IPC) was included in all AA48.48, consisting of bacterial DNA extracted from ~ 100 mg colonic content from a healthy conventional pig, 14-week-old, Danish landrace. Intestinal content was collected immediately after euthanization and frozen at – 80°C. A 10% PBS suspension was made from the intestinal content, and from here on, the protocol was identical to the one used for the reference bacteria.

DNA concentration and purity were assessed by the 260/280 nm-ratio using the Nanodrop® ND-1000 (NanoDrop Technologies Inc., Wilmington, Germany) spectrophotometer (Additional file 2). DNA was stored at -20°C until needed.

##### 

**Verifying the Gut Microbiotassay on the Access Array 48.48** Tenfold serial dilutions ranging from 0.5 pg/μl to 50 ng/μl were made from DNA extracted from 15 reference bacteria. The AA48.48 was processed following the ‘Access Array System™ User Guide’ [8]. In short: primer sets were mixed in equal concentrations and diluted to 4 μM with 20 × Access Array Loading Reagent (Fluidigm, South San Francisco, CA, USA) and nuclease-free water (Ambion Inc., Austin, USA). Master Mix was prepared as described in the instructions, and sample solutions were produced from 4 μl Master Mix and 1 μl DNA. The AA48.48 was primed in a ‘pre-PCR’ IFC controller AX (Fluidigm), before it was processed in the Biomark (Fluidigm) using the Fluidigm ‘AA 48 × 48 Standard v1’-protocol listed in Table 2. Subsequently the amplicons were harvested in a ‘post-PCR’ IFC controller AX (Fluidigm).

**Table 2:** **The Fluidigm ‘AA 48 × 48 Standard v1’ PCR thermal protocol**[8]

**PCR stages**	**Temperature**	**Number of Cycles**
Thermal mixing and hot start phase	50°C 2 minutes	
	70°C 20 minutes	1
	95°C 10 minutes	
	95°C 15 seconds	
PCR cycle	60°C 30 seconds	10
	72°C 1 minute	
C0t cycle	95°C 15 seconds	2
	80°C 30 seconds	
	60°C 30 seconds	
PCR cycle	72°C 1 minute	8
	95°C 15 seconds	
	60°C 30 seconds	
	72°C 1 minute	
C0t cycle	95°C 15 seconds	2
	80°C 30 seconds	
	60°C 30 seconds	
	72°C 1 minute	
PCR cycle	95°C 15 seconds	8
	60°C 30 seconds	
	72°C 1 minute	
C0t cycle	95°C 15 seconds	5
	80°C 30 seconds	
	60°C 30 seconds	
Extension	72°C 1 minute	1

Harvested amplicons were measured on the Agilent DNA 1000 chip (Agilent Technologies, Waldbronn, Germany) with the Agilent 2100 Bioanalyzer (Agilent Technologies) to verify the specificity of the Gut Microbiotassay. This was assessed from the size and number of amplicons generated by the primer sets listed in Table 1, with the reference bacteria as targets.

Three primer concentrations (1, 2 and 4 μM), and three primer:454BL-ratios (1:0.2, 1:0.4 and 1:0.8 μM) were tested, before settling on the final protocol.

### Testing the Gut Microbiotassay on complex samples

#### ***Samples and sampling***

To explore the efficiency range of the Gut Microbiotassay, and test its sensitivities under varying circumstances, the study included 12 three-day-old piglets from conventional pig-farms (Danish landrace); seven with and five without clinical diarrhoea (Additional file 3: Table S2). None of the animals had received any treatment. Piglets were sacrificed and luminal content from the small and large intestine were obtained immediately after and frozen at -80°C. All handling of the animals was in accordance with regulations from The Danish Centre for Animal Welfare.

#### ***DNA extraction***

100 mg intestinal content was suspended in 900 μl PBS and bead-beated in 2 ml microcentrifuge tubes containing a 5 mm steel bead (Qiagen, Hilden, Germany) at 15.0 hertz for 2.3 min (Tissuelyser II, Qiagen). Tubes were centrifuged at 10,000 × *g,* 90 s, 20°C, and 350 μl of supernatant were transferred to new tubes. These were placed in the QIAsymphony SP (Qiagen) for DNA extraction using the QIAsymphony Virus/Bacteria Mini Kit (Qiagen) with the protocol ’Pathogen complex 200′ (Qiagen), elution volume: 60 μl. DNA was measured as previously mentioned and stored at -20°C until further processing (Additional file 3: Table S2).

#### ***Analysing complex samples on the AA48.48 with the Gut Microbiotassay***

To test the amplification efficiency on DNA extracted from intestinal content ‘sample-calibration-curves’ were constructed from tenfold serial dilutions (0.25 pg/μl - 25 ng/μl). These were made from pooled sample material of 1 μl extracted DNA from each of the samples listed in Additional file 3: Table S2. ‘Control-calibration-curves’ were constructed from the IPC, and ‘reference-calibration-curves’ were generated by pooling all reference bacteria in equal concentrations.

DNA extracted from the pig samples included in the study was diluted to 50 ng/μl with nuclease-free water (Ambion). To test how the addition of a 454BL affected the Cq values, an AA48.48 with a 454BL and one without a 454BL were run under the exact same conditions. With a 454BL, each sample mixture consisted of 3 μl Master Mix, 1 μl sample (DNA, 50 ng/μl), and 1 μl Access Array Barcode Library for the 454 GS FLX Titanium Sequencer (Fluidigm), 2 μM. Afterwards the amplicons were harvested and stored at -20°C until needed.

#### ***Amplicon preparation for NGS***

To normalize the harvested sample amplicons for NGS, they were measured as described earlier and pooled in equal concentrations. This resulted in a total volume of 144.27 μl of 16 ng/μl which was purified to remove any PCR by-products. First the volume was reduced to 15 μl by extracting the DNA with phenol chloroform using a standard procedure [15]. Next, the extracted DNA was run in a 0.7% Seakem® LE Agorose gel (Lonza Rockland, Rockland, ME, USA) for 86 min, 90 V, and incubated for 30 min in 0.0004% ethidium bromide. Bands were visualized with UV-radiation using the Bio-Rad Universal hood II (Segrate, Milan, Italy) and bands in the size range 200–900 bp were excised, equalling expected amplicon sizes. DNA was extracted from the excised gel using the Qiaquick Gel Extraction Kit (Qiagen) in accordance with the kit manual.

The final pool of purified DNA from the 12 piglets to be sequenced was 723.25 ng (260/280-nm ratio: 1.96). This was run on a quarter PicoTiterPlate™ on a 454 GS FLX Titanium Sequencer (Roche) by LGC Genomics (GmbH, Berlin, Germany).

#### ***Data analysis***

##### 

**From raw Cq values to relative quantitative data** Cq values generated from the AA48.48 without a 454BL added were exported from the ‘Fluidigm Real-Time PCR Analysis software’, version 3.0.2 (Fluidigm), to Excel. Cq values were corrected to the IPC included in all runs, and those exceeding primer specific cut-off values, determined from the verification step of the Gut Microbiotassay (Additional file 4: Table S3), were excluded. Relative quantification was calculated from the mean of the technical replicates using the Livak-method [16]. This method was chosen based on the theory that the total amount of bacteria, targeted by the primer set domain Bacteria B, constituted 100% of the microbiota in the individual gut section at all times. Hence, the Cq values of all primer sets for each sample were normalised against the Cq value of their respective domain Bacteria B primer set: $R_{\left(\mathrm{primer}\;\mathrm{set}\;X\right)}=2^{Cq_{\left(\mathrm{domain}\;\mathrm{Bacteria}\;B\right)}-Cq_{\left(\mathrm{primer}\;\mathrm{set}\;X\right)}}$. To compare total bacteria detected with the domain Bacteria B primer set, these Cq values were related to the final number of thermal cycles run, 35: $R_{\left(\mathrm{domain}\;\mathrm{Bacteria}\;B\right)}=2^{35-Cq_{\left(\mathrm{domain}\;\mathrm{Bacteria}\;B\right)}}$. Normalization was further done to total mean of all primer sets for each sample with similar results (data not shown).

##### 

**454-sequencing data** Sequence data, available at NCBI Sequence Read Archive under Accession SRA061551, was analysed using BION, a yet unpublished open source program. For more information on the BION software, its functions, and the main statistics for the raw results see Additional file 5. In short: the sequence dataset was converted to FASTQ, split according to sample barcodes and primer sequences, and trimmed in the same process. Next, sequences were cleaned using a cut-off for minimum quality of 96%, and a minimum sequence length of 200 bp. The remaining sequences were clustered using UCLUST, based on a minimum seed similarity of 99.5%. Query sequences were compared to Greengenes Gene Database [17] using the k-mer matching program Simrank2, an improved version of Simrank [18]. Simrank2 returns the n % best similarities, no matter how low they are. Also, it only produces 8-mers from regions above a given quality, and skips sequences with too few 8-mers. This last feature helps improve the data quality. Simrank2 was set to return the best 1% similarities with a similarity cut-off of 50%. Taxonomy was generated by transforming percentages for the Greengenes OTUs to scores, read densities, the sum of which was 1. This was done in a weighted manner, so that OTUs of the highest similarity scored a high number and vice versa, the sum of which was the original reads. Only phylotypes with a primer-specific read density of ≥ 1% were included in the statistical analysis.

##### 

**Statistics** Initially, all data were transformed with the natural logarithm. Primer sets with more than half of the data missing were removed from the dataset. If primer sets in taxonomic lineage showed pair-wise correlations above 0.99, only the primer set of the highest taxonomic level in the lineage was retained for analysis, and any conclusion drawn from this also accounted for the excluded sub-level primer sets. To include the information from remaining data-deficient primer sets, for which the fraction of missing data was low and never above 0.35, the EM-algorithm [19] was used to substitute missing values with imputed ones, by applying a multivariate Gaussian model. Each primer set was allowed to depend freely on the others and also for dependency on gut section, diarrhoeic status, and interaction between these. Model fit of the multivariate model for the primer sets after imputation was assessed by transforming the model residuals with the inverse of the square root of the estimated covariance matrix between the primer sets, and applying standard model control to these standardized residuals. These analyses were consistent with standard model behaviour.

Effect of gut section and diarrhoeic status was tested with the Likelihood Ratio Method using the Wilks test [20]. Sequence data were compared for effect of diarrhoeic status using the non-parametric Wilcoxon test [21].

P-values < 0.05 were considered statistically significant.

## Results

### Designing the Gut Microbiotassay

The Gut Microbiotassay was constructed from 49 primers constituting 24 primers sets (Table 1) in order to target the main bacterial Phyla (Actinobacteria, Bacteroides, Firmicutes, Fusobacteria, Proteobacteria and Verrucomicrobia) and some of the highly important bacterial groups and species reported in the mammalian intestinal tract [9]. 12 primers were designed *de novo*, and 37 primers were from published literature, of which ten were modified. Five of the 24 primer sets were unmodified pairs from published literature, whereas the rest were combinations composed in this study. As it was not possible to find or design specific primer sets targeting the 16S rRNA gene for class β- and γ-proteobacteria, and genus *Lactobacillus,* these were designed to target the 23S rRNA gene instead.

### Sensitivity and specificity of the Gut Microbiotassay

**Figure 1:**
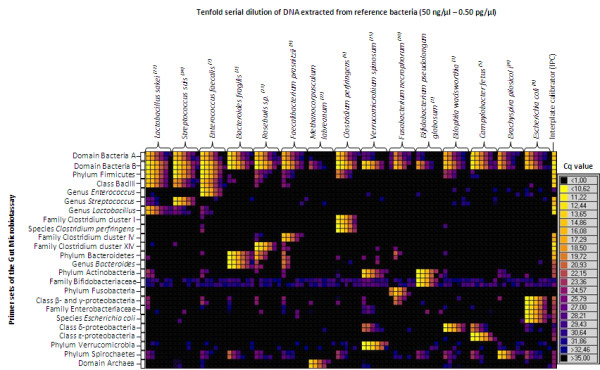
**Heatmap generated by the Fluidigm Real-Time PCR Analysis program from raw Cq data.** At the top horizontal: Tenfold serial dilutions of DNA (ranging from 50 ng/μl down to 0.5 pg/μl) extracted from 15 different reference bacteria. Raised numbers in parentheses represent the respective numbers given to each reference bacterium in Table 1. These were used to test the specificity and sensitivity of the primer sets included in the Gut Microbiotassay, listed vertically on the left. Primers were run in duplicates. On the right: a colour scale depicting the respective Cq values (the software uses commas instead of points as decimal separator for Cq values).

Tenfold serial dilutions of DNA extracted from 15 reference bacteria were used to evaluate the specificity and sensitivity of the Gut Microbiotassay (Figure 1).

The specificity for each primer set was assessed from the Cq values obtained for their respective target bacteria in addition to any cross reaction. For 13 of the 24 primer sets, specific positive reactions were registered on various taxonomic levels in agreement with the reference bacteria listed for each primer set in Table 1. The remaining primer sets showed different degrees of cross reaction (Figure 1). On average, the lowest unspecific and the highest specific Cq of same concentration differed by 9 Cq values, equalling less than 1% cross reaction. Apparently, the primer set phylum Firmicutes did not have a complete coverage, as it only amplified few bacteria from the Clostridia clusters. The highest specific Cq value was determined for each primer set from the representative target bacterial species and used as cut-off value in the data analysis (Additional file 4: Table S3).

The dynamic range for the specific primer sets spanned from 50 ng to 50 pg DNA/μl for two primer sets, to 5 pg DNA/μl for 15 primer sets, and for seven primer sets down to 0.5 pg DNA/μl (lowest concentration tested), Table 3. Linear regression of log-concentration versus Cq values for the different primer sets demonstrated *r*^*2*^ from 0.943 to 0.999 (SD 0.0074). Gel pictures and electropherograms confirmed specific amplification by demonstrating the expected number of amplicons corresponding to the number of taxonomic levels represented by the specific primer sets. Additionally, amplicon sizes (bp) were comparable to those listed in Table 1.

**Table 3:** **The primer efficiency, *r***^***2***^**-value, dynamic range and limit of detection of the Gut Microbiotassay**

**Primer set**	**Efficiency, %**	***r***^***2***^	**Dynamic range**	**Limit of detection, ng/μl**
Domain Bacteria B V4-V5	75-106	>0.968	4-6-fold	0.05-0.0005
Phylum Firmicutes*	86-97	>0.994	3-5-fold	0.05-0.005
Phylum Bacilli	99-101	>0.943	4-5-fold	0.05-0.005
Genus *Enterococcus*	87	>0.998	5-fold	0.005
Genus *Lactobacillus*	112	>0.992	5-fold	0.005
Genus *Streptococcus*	102	>0.999	5-fold	0.005
Family Clostridium cluster I	96	>0.995	4-fold	0.05
Species *Clostridium perfringens*	87	>0.999	5-fold	0.005
Family Clostridium cluster IV	103	>0.997	5-fold	0.005
Family Clostridium cluster XIV	96	>0.999	4-fold	0.05
Phylum Bacteroidetes	91	>0.991	6-fold	0.0005
Genus *Bacteroides*	85	>0.997	5-fold	0.005
Phylum Actinobacteria	92	>0.988	6-fold	0.0005
Family Bifidobacteriaceae	77	>0.995	5-fold	0.005
Class β- and γ-proteobacteria	87	>0.999	5-fold	0.005
Family Enterobacteriacea	84	>0.996	5-fold	0.005
Species *Escherichia coli*	91	>0.999	6-fold	0.0005
Class ϵ-proteobacteria	86	>0.995	6-fold	0.0005
Class δ-proteobacteria	91	>0.995	5-fold	0.005
Phylum Fusobacteria	85	>0.997	4-fold	0.005
Phylum Verrucomicrobia	107	>0.990	6-fold	0.0005
Phylum Spirochaetes	80	>0.996	5-fold	0.005
Domain Archaea	95	>0.996	5-fold	0.005

*Data from the reference bacteria *Roseburia sp.* and *F. Prausnitzii* has been excluded in primer set Phylum Firmicutes.

The Gut Microbiotassay was tested against tenfold dilution series of DNA extracted from individual reference bacteria without a 454 Barcode Library added.

Fluidigm’s recommendation on DNA sample concentration is 25–50 ng/μl [8]. When testing the amplification efficiency on 23 calibration curves (ranges: 0.25 pg/μl – 50 ng/μl), there was no PCR inhibition for either of the primer sets with DNA of 25 or 50 ng/μl.

#### ***Addition of a 454 Barcode Library***

Including a 454BL with the reactions did not affect the Cq values drastically. The biggest deviation was less than 1 Cq compared to the respective Cq values without a 454BL added, and 90 percent of the corresponding values from the two data sets were correlated by *r*^*2*^ > 0.90. Uncorrelated data were mainly seen if no or very few target bacteria were present in the reaction. In such cases the 454BL occasionally caused some interaction with itself or/and the present primer set, creating a signal that obscured the low or missing signal from the target bacteria. Because of this, samples were run twice: with and without a 454BL added. Cq values without a 454BL were used for data analysis, whereas amplicons with the 454BL incorporated were available for 454-sequencing.

### The Gut Microbiotassay on complex samples

Piglets of different diarrhoeic status were sacrificed and luminal content was collected from the small and the large intestine. This resulted in a total of 23 samples divided into the following groups: small intestine without diarrhoea (S-) n = 5, small intestine with diarrhoea (S+) n = 7, large intestine without diarrhoea (L-) n = 4, and large intestine with diarrhoea (L+) n = 7.

#### ***AA48.48 general findings***

The following primer sets were missing more than half of the data, and were consequently removed from the statistical data analysis: family Clostridium cluster IV, class ϵ-proteobacteria and δ-proteobacteria, phyla Verrucomicrobia, and domain Archeae. Primer sets showing pair-wise correlation above 0.99, were: class β- and γ- proteobacteria with family Enterobacteriacea, and species *E. coli*; and phylum Bacteroidetes with genus *Bacteroides*. Of the general bacteria primers only domain Bacteria B was tested as this was the primer set used for normalization. This left 15 primer sets for data analysis. The multivariate test for effect of gut section and diarrhoeic status, with effects allowed to differ with the values of gut section, revealed statistically significant effects (S/L *p* = 0.01, S+/S - *p* = 0.002, and L+/L - *p* = 0.006, respectively). However, effects were not limited to the primer sets that showed significant differences in the Gut Microbiotassay. If these were excluded, and the data analysis repeated, this still resulted in a significant effect from diarrhoeic status in the large intestine (L+/ L - *p* = 0.01), while it was borderline insignificant in the small intestine (S+/ S - *p* = 0.056).

Table 4 lists the estimated mean percentages and 95% confidence interval for each primer set included in the data analysis relative to total bacteria. Diarrhoeic gut sections contained fewer bacteria from phylum Firmicutes (genus Streptococcus and unclassified (*p* < 0.05)), but a higher fraction of genus *Enterococcus* (small intestine *p* = 0.04). There was a highly significant depletion of members from genus *Streptococcus* in the diseased compared to the healthy intestines. The same tendency was seen for Clostridium cluster I (small intestine *p* = 0.02). The diarrhoeic small intestine harboured fewer members from class β- and γ-proteobacteria than the healthy one, while this was reversed for the large intestine (*p* < 0.05). Generally healthy piglets had a gut microbiota dominated by Gram-positive bacteria, which was partly displaced by Gram-negative bacteria in diarrhoeic piglets.

**Table 4:** Estimated mean primer set values from the AA48.48 and the corresponding significant 454 GS FLX Titanium sequencing results

**Primer set**	**Estimated mean percentages relative to total bacteria**				**P-values**	
	**S-**	**S+**	**L-**	**L+**	**S+/ S-**	**L+/ L-**
**Domain Bacteria B**^**1**^	24.96	20.32	25.13	25.46	**0.003**	0.857
	[22.39;27.73]	[16.83;24.32]	[22.26;28.27]	[23.23;27.84]		
Genus *Streptococcus*					**0.0061**	**0.0061**
Species *S. alactolyticus*					**0.0099**	**0.0134**
**Phylum Firmicutes**	36.08	27.59	30.59	17.79	0.649	0.278
	[17.64;65.91]	[7.50;72.58]	[13.64;59.56]	[9.79;29.83]		
Genus *Streptococcus*					**0.0242**	**0.0102**
Unclassified					**0.0462**	**0.0176**
**Class Bacilli**	14.01	15.92	8.30	12.04	0.473	0.52
	[4.85;32.09]	[2.22;57.40]	[2.50;20.64]	[4.99;24.63]		
Genus *Streptococcus*					**0.0287**	**0.0061**
Unclassified					0.1516	**0.029**
**Genus *Enterococcus***	0.036	0.39	0.04	0.11	0.208	0.34
	[0.0038;0.14]	[0.0049;2.51]	[0.0032;0.18]	[0.018;0.39]		
Genus *Enterococcus*					**0.0424**	0.9273
**Genus *Streptococcus***	4.04	1.00	5.10	0.56	**0.0003**	**0.00002**
	[1.84;7.73]	[0.24;2.83]	[2.10;10.46]	[0.29;0.98]		
Genus *Streptococcus*					**0.0061**	**0.0121**
Species *S. alactolyticus*					**0.0467**	**0.0121**
**Genus *Lactobacillus***	33.97	53.70	33.27	27.55	0.643	0.846
	[12.03;76.74]	[7.82;190.09]	[10.27;81.52]	[11.62;55.67]		
**Family Clostridium cluster I**	0.65	0.17	0.32	0.079	0.231	0.139
	[0.16;1.81]	[0.012;0.76]	[0.064;0.97]	[0.025;0.19]		
Genus *Clostridium*					**0.0171**	0.2558
**Species C** ***lostridium perfringens***	3.03	2.82	2.33	0.63	0.81	0.219
	[0.62;9.24]	[0.14;14.31]	[0.38;7.86]	[0.17;1.66]		
**Family Clostridium cluster XIV**	0.89	16.84	10.82	5.02	0.818	0.672
	[0.098;3.53]	[0.22;106.79]	[0.86;47.63]	[0.82;16.95]		
**Phylum Bacteroidetes and Genus Bacteroides**	2.25	76.17	22.05	5.10	0.924	0.567
	[0.082;12.19]	[0.091;505.28]	[0.48;130.66]	[0.34;23.65]		
Species *Bacteroides uniformis* (generated from the primer set Genus Bacteroides)					**0.0447**	0.0985
**Phylum Actinobacteria**	0.32	3.92	1.02	0.32	**0.034**	0.068
	[0.13;0.68]	[0.70;12.70]	[0.35;2.32]	[0.15;0.61]		
Genus *Actinomyces*					1	**0.0363**
Species *Actinomyces hyovaginalis*					0.1042	**0.0179**
Species *Bifidobacterium pseudolongum*					1	**0.0368**
Unclassified					0.072	**0.0104**
**Family Bifidobacteriaceae**	0.0064	0.011	0.0063	0.012	0.399	0.24
	[0.0026;0.013]	[0.0022;0.036]	[0.0023;0.014]	[0.0056;0.022]		
Species *Bifidobacterium pseudolongum*					1	**0.0424**
**Phylum Fusobacteria**	1.59	32.60	6.28	9.71	0.413	0.604
	[0.18;6.17]	[0.48;203.87]	[0.54;27.06]	[1.66;32.13]		
**Class β- and γ-proteobacteria; Family Enterobacteriaceae and Species *Escherichia coli***	2.96	0.54	0.61	3.17	0.6	**0.029**
	[0.83;7.69]	[0.049;2.28]	[0.14;1.72]	[1.10;7.25]		
Family Enterobacteriaceae (generated from the primer set Family Enterobacteriaceae)					**0.0424**	1
Species *Escherichia fergusonii* (generated from the primer set Family Enterobacteriaceae)					**0.0368**	0.7758
Genus *Shigella* (generated from the primer set Family Enterobacteriaceae)					**0.0462**	0.9244
Species *Shigella flexneri* (generated from the primer set Family Enterobacteriaceae)					**0.0413**	0.5626
**Phylum Spirochaetes**	0.0059	0.010	0.0091	0.0056	0.73	0.081
	[0.004;0.0085]	[0.005;0.019]	[0.0058;0.014]	[0.004;0.0076]		
Genus *Streptococcus*					**0.0169**	**0.0127**
Unclassified					**0.0191**	0.0694

^1^Domain Bacteria B is expressed in percentage relative to the final number of thermal cycles run, 35.

Primer set values from the AA48.48 are expressed as percentages relative to total bacteria, and beneath (indented) are listed the significant 454-sequencing results for the respective primer sets. Numbers in bold indicate significant p-values. L: Large intestine, S: small intestine, +: with diarrhoea, –: without diarrhoea. [95% lower level; 95% upper level].

#### ***454-Sequencing results***

**Figure 2:**
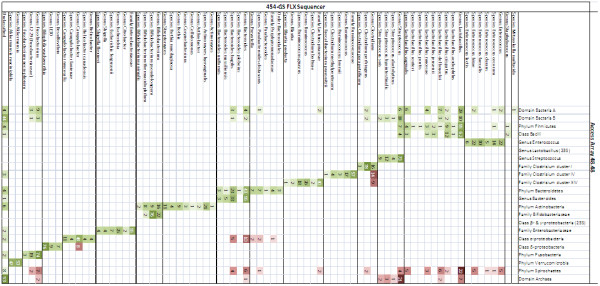
**Specificity of the Gut Microbiotassay.** The figure shows the sequencing results and corresponding read density scores (≥ 1%) for the individual primer sets of the Gut Microbiotassay targeting the 16S rRNA gene. Green colours indicate sequences corresponding to the primer sets’ target, whereas red colours represents unspecific amplification. The darker the colour, the higher the read density score.

Sequencing barcoded sample amplicons resulted in 275,133 unprocessed consensuses, which dropped to 164,055 after quality trimming. Amplicons generated by the primer sets domain Bacteria A and B encompassed 16S rRNA gene sequences from both Gram-positive and Gram-negative bacteria genera plus from unclassified bacteria. Good congruency was found between the primer sets of the Gut Microbiotassay and the 454-sequencing results generated from their respective amplicons (Figure 2).

#### ***Comparing significant findings from AA48.48 with 454-sequencing data***

Based on the Gut Microbiotassay data for the individual bacterial groups, four groups showed significant differences: domain Bacteria B, genus *Streptococcus,* phylum Actinobacteria, and class β- and γ-proteobacteria, Table 4. In the small intestine, diarrhoea was associated with a significantly reduced bacterial load (*p =* 0.003). Sequence data generated by the domain Bacteria B primer set indicated that diarrhoea was associated with a decreased number of members from genus *Streptococcus,* including *S. alactolyticus* in both the small intestine (*p =* 0.0061, and *p =* 0.0099, respectively) and the large intestine (*p =* 0.0061 and *p =* 0.013). When comparing the results for genus *Streptococcus*, there were significantly fewer Streptococci in the diseased intestines (S+/ S - *p* = 0.0003, L+/ L - *p* = 0.00002). The sequences generated by genus *Streptococcus* confirmed the previous results (S+/ S - *p* = 0.0061, L+/ L - *p* = 0.012). These were classified to: genus *Streptococcus*, species *S. hyointestinalis, S. suis*, and *S. alactolyticus.* However, at species level, only *S. alactolyticus,* showed coherently significant results with genus *Streptococcus* (S+/ S - *p* = 0.047, L+/ L - *p* = 0.012). Phylum Actinobacteria was more abundant in the diarrhoeic compared to the healthy small intestine (*p* = 0.034), however, this was not reflected in the sequencing data for the small intestine. The diarrhoeic large intestine possessed significantly more members from the class β- and γ-proteobacteria, including family Enterobacteriaceae and species *E. coli*, than the healthy one (*p* = 0.029). Nonetheless, for this gut section no significant differences showed up in the sequencing data from the family Enterobacteriaceae primer set.

#### ***Specificity of the Gut Microbiotassay***

After sequencing the amplicons generated by the Gut Microbiotassay the specificity of the primer sets was revaluated (Figure 2), except for the primer sets targeting the 23S rRNA gene (genus *Lactobacillus* and class β- and γ-proteobacteria) as the freeware used to analyze the sequence data is currently based on 16S rRNA gene databases.

16 of the 24 primer sets only generated sequences from their intended target group, four showed cross reaction with a single group, and two primer sets cross reacted with more than two other taxonomic groups. The primer set phylum Firmicutes did not reveal any clostridia, as predicted earlier. In conclusion, the sequencing data confirmed the specificity of the primer sets found in the verification process of the Gut Microbiotassay when tested against DNA extracted from pure-cultured reference bacteria.

## Discussion

Most qPCR studies describing the gut microbiota typically do this by using a general bacteria primer and a few group-specific ones [4,22]. In contrast, this study developed the Gut Microbiotassay, an assay composed of 24 primer systems, capable of screening the microbiota for the most common bacteria in the mammalian intestine [9,10] at various taxonomic levels. The Gut Microbiotassay was tested against representative reference bacteria, and next on complex intestinal samples from piglets of different diarrhoeic status. The sample amplicons were harvested and sequenced, and functioned as a proof of concept, evaluated the specificity of the Gut Microbiotassay by further elucidating the components of the gut microbiota.

This approach offers an alternative to current molecular methods employed to characterize the gut microbiota such as phylogenetic microarrays [23], and NGS [24]. In contrast to phylogenetic microarrays, the AA48.48 is highly flexible because primer sets can readily be replaced to meet the needs of a current research study. In addition, the AA48.48 outmatches the phylogenetic microarray on sample capacity, as well as sensitivity [25]. Also, no pre-amplification is needed when running the Gut Microbiotassay with the AA48.48, which reduces the workload, and also the risk of introducing technical variation. The effect of such technical variation can be reduced by normalization. In the present study each sample were normalized against the Cq value of their respective domain Bacteria B primer set. Impact of normalization against their respective domain Bacteria B was tested by performing a second normalization procedure; at this point data was also normalized to total mean of all primer sets for each sample individually, with similar end results (data not shown). As the choice of normalization in the present study (Domain Bacteria B) or total mean did not have a great impact on final results we assume that the technical variation was low or that we managed to normalize efficiently using both methods. Further, data from domain Bacteria A and domain Bacteria B were highly correlated also pointing to domain Bacteria B as a reasonable reference primer efficiency of qPCR is of great importance and should ideally be within the range of 85% to 110%. The majority of the primers used in the Gut Microbiotassay (21 out of the 23 primers included in Table 3) had and acceptable efficiency between 80-110%. However, the primer Doman bacteria A was found to differ to much with regards to efficiency and dynamic range, thus data from this primer pair was only used to support data generated from the primer Doman bacteria B. Likewise primer set Phylum Firmicutes produced to high efficiency when tested on two reference bacteria *Roseburia sp.* and *F. Prausnitzii.* However *Roseburia sp.* and *F. Prausnitzii* are covered by several other well performing primers including Clostridium cluster XIV and Clostridium cluster IV respectively. Therefore the *Gut Microbiotassay* cannot be used as an absolute quantitative assay across the different primer sets. In order to make it a truly quantitative assay it will be necessary either to have a defined start sample regarding species composition or only to use specific primers on the array where high efficiency has been proven.

The heatmap generated by the software ‘Fluidigm Real-Time PCR Analysis’ (Fluidigm Corporation) depicts the raw Cq values for each reaction. This makes it feasible to quickly evaluate and visually compare the bacterial profiles across a large number of samples. As sample amplicons are harvested individually following qPCR, it is possible to pinpoint which samples to sequence for further taxonomic information. Selective sequencing reduces costs compared to non-selective sequencing. Also, the dataset generated from sequencing the Gut Microbiotassay PCR amplicons produces a much more manageable dataset compared to metagenomic approaches. The Gut Microbiotassay provides a quantitative picture of the distribution of the known gut microbiota represented by the primers. Moreover, if combined with 454-sequencing, it enables detection of bacteria with unidentified sequences [25]. A limitation of the assay when running on complex bacterial samples is that the primers will have different efficiencies and dynamic ranges due to imperfect matches with some of the target sequences. The Gut Microbiotassay has therefore the most value for analysing high-throughput quantification of the bacterial composition in many samples or samples with defined biomarkers.

The validation of the Gut Microbiotassay by sequencing the amplicons from intestinal content of two different gut sections from piglets of different diarrhoeic status demonstrated the potential to further elucidate the components of the gut microbiota. This study used the results from the Gut Microbiotassay to quantify the taxonomical groups, and NGS to access the bacterial constituents.

Common intestinal bacteria in the neonatal piglet include members of: Clostridia, Streptococcaceae, Lactobacillaceae, Enterobacteriaceae, Fusobacteria and sometimes Bacteroidetes [26,27]. These bacterial groups were also found in the gut microbiota of three-day-old piglets using the Gut Microbiotassay. The Gut Microbiotassay indicated that a healthy gut microbiota was dominated by Gram-positive bacteria, which were partly replaced by Gram-negative bacteria in the large intestine of diarrhoeic piglets. Robinson et al. [28] came to a similar conclusion in a study investigating the intestinal microbiota of pig colons experimentally induced with swine dysentery. Consistent significant findings from the Gut Microbiotassay and the 454-sequencing results implied that diarrhoea was associated with a depletion of members from the genus *Streptococcus*, and previous research has shown that Streptococci is an important member of a healthy gut microbiota [26,28,29]. A detailed review of the aetiology behind the piglet diarrhoea is beyond the scope of this paper and prevented by the limited number of piglets analysed, as the primary focus of this paper has been on the verification and application of the Gut Microbiotassay.

## Conclusions

The Gut Microbiotassay offers affordable quantitative screening of the microbiota with the AA48.48. It has been thoroughly tested and strict criteria for data analysis have been outlined. It provides a high sample capacity, a wide dynamic range, and it facilitates selective 454-sequencing afterwards. Hence, it is timesaving and economical due to the easy library preparation, the low consumption of master mix, and the optional selective sequencing. These features make the Gut Microbiotassay a worthy high-throughput competitor to the current alternative methods used for investigating diverse ecosystems.

## Abbreviations

AA48.48: Access array 48.48; 454BL: 454 Barcode library; IPC: Interplate calibrator; L: Large intestine; L+: Large intestine with diarrhoea; L-: Large intestine without diarrhoea; NGS: Next generation sequencing; S: Small intestine; S+: Small intestine with diarrhoea; S-: Small intestine without diarrhoea.

## Competing interests

The authors declare that they have no competing interests.

## Authors’ contributions

MLHB designed the Gut Microbiotassay, conducted all the experiments, and wrote the manuscript. KS supervised the study and contributed to the origin of this manuscript. AS performed the statistical calculations and provided all information on statistical issues. NL analysed the 454-sequence data using his BION software. LM has been the primus of this project and has guided and supervised the progress of both the project and the manuscript. All authors read and approved the final manuscript.

## Supplementary Material

Additional file 1: Table S1 Concentration and purity of DNA extracted from the reference bacteria and the interplate calibrator. Click here for file

Additional file 2 Protocol used for DNA extraction with the Easy-DNA™ Kit (Invitrogen, Carlsbad, CA, USA). Click here for file

Additional file 3: Table S2 Piglets included in the study. Click here for file

Additional file 4: Table S3 Cross reactions detected between primer systems and reference bacteria tested. Highest specific Cq value determined from the respective target reference bacteria has been used as cut-off value for the different primer systems in the data analysis. Click here for file

Additional file 5 **BION analysis of 454-sequencing data.** Detailed information on the BION software, its functions, and the main statistics for the raw results is included in the file Results_referee.zip. When the file is unpacked it creates the directory Results_referee. This contains 24 subfolders containing the main statistics for each primer pair. However, the species-folders are empty, since the species-specific primers were not tagged. The remaining subfolder ‘Software’ and files are explained in the README-file. The entire BION-meta package (200 Mb) is not included, and there is currently not a stable link to it. But if interested, it can be downloaded from the following link: https://www.dropbox.com/sh/fumscuqpanqaqvu/_4H--XBxHQ. Click here for file
