# Supplementary material for: The Gut Microbiotassay: a high-throughput qPCR approach combinable with next generation sequencing to study gut microbial diversity
Source: BMC Genomics. 2013 Nov 14;14:788. doi: 10.1186/1471-2164-14-788 (PMC3879714; doi:10.1186/1471-2164-14-788)
Supplement: Additional file 1: Table S1 — Concentration and purity of DNA extracted from the reference bacteria and the interplate calibrator. [file 1471-2164-14-788-S1.doc]

**Additional file 1:** **Concentration and purity of DNA extracted from the reference bacteria and the interplate calibrator.**

| **Reference bacterium** | **Concentration, ng/μl** | **260/280 nm-ratio** |
| --- | --- | --- |
| *Bacteroides fragilis* (DJF_B083(EU728706)) | 1728.16 | 2.21 |
| *Bifidobacterium pseudolongum globosum* (DMS 20092) | 58.34 | 2.17 |
| *Bilophila wadsworthia* (ATCC 49260) | 29.39 | 2.07 |
| *Brachyspira pilosicoli* (isolated from intestine of slaughter pig at DTU-VET, 28-02-2000) | 487.44 | 2.18 |
| *Campylobacter fetus* (ATTC 10852) | 232.53 | 2.2 |
| *Clostridium perfringens* (NCTC 10240) | 417.35 | 2.21 |
| *Enterococcus faecalis* (ATCC 29212) | 233.57 | 2.22 |
| *Escherichia coli* (9711108-2) | 502.74 | 2.15 |
| *Faecalibacterium prausnitzii* (DSM 17677) | 190.59 | 2.17 |
| *Fusobacterium Necrophorum* (ATCC 25286) | 27.9 | 1.71 |
| *Lactobacillus sakei* (DSM 20017) | 219.39 | 2.28 |
| *Methanocorpusculum labreanum* (DSM 4855) | 21.26 | 1.69 |
| *Roseburia sp.* (DJF_VR77(EU728794)) | 85.56 | 2.17 |
| *Streptococcus suis* (NCTC 10446) | 63.19 | 2.17 |
| *Verrucomicrobium spinosum* (DSM 4136) | 65.84 | 1.97 |
| Interplate calibrator (IPC) | 701.84 | 2.06 |
