# Supplementary material for: The Gut Microbiotassay: a high-throughput qPCR approach combinable with next generation sequencing to study gut microbial diversity
Source: BMC Genomics. 2013 Nov 14;14:788. doi: 10.1186/1471-2164-14-788 (PMC3879714; doi:10.1186/1471-2164-14-788)
Supplement: Additional file 2 — Protocol used for DNA extraction with the Easy-DNA™ Kit (Invitrogen, Carlsbad, CA, USA). [file 1471-2164-14-788-S2.doc]

**Additional file 2**

***Protocol used for DNA extraction with the Easy-DNA™ Kit (Invitrogen, Carlsbad, CA, USA)***

Pure-cultured colonies were suspended in 1000 μl PBS, and 175 μl of this was mixed with 250 μl Solution A, and incubated at 65 °C for 6 min. 450 μl chloroform was added to the lysate , vortexed until homogenised, and 100 μl of Solution B were transferred to each suspension and vortexed. After centrifuging the suspensions for 10 min, 10 000 *g*, 15 °C, the upper liquid phase containing the DNA was decanted to new tubes. The DNA was precipitated with 5 μl of 5 M NaCl and 1 ml of ice-cold 96% ethanol; next, tubes were centrifuged for 45 min, 20 000 *g,* 4 °C. Liquid was removed and 1 ml of ice-cold 70% ethanol was added, followed by centrifugation for 10 min, 20 000 *g,* 4 °C. The ethanol was discarded and the DNA-pellet was resuspended in 60 μl TE buffer.
