# Supplementary material for: The Gut Microbiotassay: a high-throughput qPCR approach combinable with next generation sequencing to study gut microbial diversity
Source: BMC Genomics. 2013 Nov 14;14:788. doi: 10.1186/1471-2164-14-788 (PMC3879714; doi:10.1186/1471-2164-14-788)
Supplement: Additional file 3: Table S2 — Piglets included in the study. [file 1471-2164-14-788-S3.doc]

**Additional file 3**: **Piglets included in the study. All piglets were three days old.**

| **Ear tag** | **Diarrhoea** | **Barcode** | | **Gut section** | **DNA measurements** | |  |
| --- | --- | --- | --- | --- | --- | --- | --- |
|  |  | **MID^1^** | **Sequence^2^** |  | **Concentration, ng/μl** | **260/280 nm-ratio** | |
| **83183-1** | yes | MID-16 | TCACGTACTA | large | 224.45 | 1.94 | |
|  |  | MID-17 | CGTCTAGTAC | small | 26.15 | 2.27 | |
| **83183-2** | yes | MID-18 | TCTACGTAGC | large | 50.57 | 2.07 | |
|  |  | MID-19 | TGTACTACTC | small | 120.08 | 1.98 | |
| **83183-3** | yes | MID-20 | ACGACTACAG | large | 60.71 | 2.06 | |
|  |  | MID-21 | CGTAGACTAG | small | 31.85 | 2.11 | |
| **83183-4** | yes | MID-22 | TACGAGTATG | large | 51.6 | 2.05 | |
|  |  | MID-23 | TACTCTCGTG | small | 81.24 | 1.98 | |
| **83183-5** | yes | MID-24 | TAGAGACGAG | large | 24.39 | 2.2 | |
|  |  | MID-25 | TCGTCGCTCG | small | 186.96 | 1.96 | |
| **83244-1** | yes | MID-35 | CAGTAGACGT | large | 29.5 | 2.16 | |
|  |  | MID-36 | CGACGTGACT | small | 104.26 | 2.01 | |
| **83244-2** | yes | MID-37 | TACACACACT | large | 44.7 | 2.08 | |
|  |  | MID-38 | TACACGTGAT | small | 43.39 | 2.17 | |
| **83184-1** | no | MID-26 | ACATACGCGT | large | 57.43 | 2.07 | |
|  |  | MID-27 | ACGCGAGTAT | small | 41.22 | 2.13 | |
| **83184-2** | no | MID-28 | ACTACTATGT | large | 43.58 | 2.11 | |
|  |  | MID-29 | ACTGTACAGT | small | 82.5 | 1.95 | |
| **83184-3** | no | MID-30 | AGACTATACT | large | 89.85 | 1.98 | |
|  |  | MID-31 | AGCGTCGTCT | small | 46.01 | 2.09 | |
| **83184-4** | no | MID-32 | AGTACGCTAT | small | 35.82 | 2.23 | |
| **83184-5** | no | MID-33 | ATAGAGTACT | small | 47.67 | 2.14 | |
|  |  | MID-34 | CACGCTACGT | large | 36.19 | 2.09 | |

**^1^** MID: Multiplexing Identifier, **^2^** Access Array Barcode Library for the 454 GS FLX Titanium Sequencer (Fluidigm, South San Francisco, CA, USA).
