# Supplementary material for: The Gut Microbiotassay: a high-throughput qPCR approach combinable with next generation sequencing to study gut microbial diversity
Source: BMC Genomics. 2013 Nov 14;14:788. doi: 10.1186/1471-2164-14-788 (PMC3879714; doi:10.1186/1471-2164-14-788)
Supplement: Additional file 4: Table S3 — Cross reactions detected between primer systems and reference bacteria tested. Highest specific Cq value determined from the respective target reference bacteria has been used as cut-off value for the different primer systems in the data analysis. [file 1471-2164-14-788-S4.doc]

**Additional file 4:** **Cq cut-off values:** The highest specific Cq value was determined from the respective target reference bacteria and used as cut-off value for the different primer systems in the data analysis.

| **Primer system** | **Cq cut-off value** |
| --- | --- |
| Domain Bacteria A V2-V3 | 31.45 |
| Domain Bacteria B V4-V5 | 28.04 |
| Phylum Firmicutes | 30.03 |
| Phylum Bacilli | 31.33 |
| Genus *Enterococcus* | 24.59 |
| Genus *Lactobacillus* | 28.80 |
| Genus *Streptococcus* | 24.30 |
| Family Clostridium cluster I | 26.30 |
| Species *Clostridium perfringens* | 24.35 |
| Family Clostridium cluster IV | 24.10 |
| Family Clostridium cluster XIV | 25.85 |
| Phylum Bacteroidetes | 26.08 |
| Genus *Bacteroides* | 29.60 |
| Phylum Actinobacteria | 21.55 |
| Family Bifidobacteriaceae | 26.21 |
| Class β- and γ-proteobacteria | 24.45 |
| Family Enterobacteriacea | 25.48 |
| Species *Escherichia coli* | 25.32 |
| Class ε-proteobacteria | 23.95 |
| Class δ-proteobacteria | 26.03 |
| Phylum Fusobacteria | 22.82 |
| Phylum Verrucomicrobia | 21.91 |
| Phylum Spirochaetes | 25.28 |
| Domain Archaea | 23.53 |
