# Supplementary material for: The Gut Microbiotassay: a high-throughput qPCR approach combinable with next generation sequencing to study gut microbial diversity
Source: BMC Genomics. 2013 Nov 14;14:788. doi: 10.1186/1471-2164-14-788 (PMC3879714; doi:10.1186/1471-2164-14-788)
Supplement: Additional file 5 — BION analysis of 454-sequencing data. Detailed information on the BION software, its functions, and the main statistics for the raw results is included in the file Results_referee.zip. When the file is unpacked it creates the directory Results_referee. This contains 24 subfolders containing the main statistics for each primer pair. However, the species-folders are empty, since the species-specific primers were not tagged. The remaining subfolder ‘Software’ and files are explained in the README-file. The entire BION-meta package (200 Mb) is not included, and there is currently not a stable link to it. But if interested, it can be downloaded from the following link: https://www.dropbox.com/sh/fumscuqpanqaqvu/_4H--XBxHQ. [file 1471-2164-14-788-S5.doc]

**Additional file 5**

***BION analysis of 454-sequencing data***

Detailed information on the BION software, its functions, and the main statistics for the raw results is included in the file Results_referee.zip. When the file is unpacked it creates the directory Results_referee. This contains 24 subfolders containing the main statistics for each primer pair. However, the species-folders are empty, since the species-specific primers were not tagged. The remaining subfolder ‘Software’ and the additional files are explained in the README-file.

The entire BION-meta package (200 Mb) is not included, and there is currently not a stable link to it. But if interested, it can be downloaded from the following link:

<https://www.dropbox.com/sh/fumscuqpanqaqvu/_4H--XBxHQ>
